# Chitosan-Based Colloidal Delivery Systems as Natural Carriers for Propolis: Physicochemical Characterization and Application in Walnut Sausages

**DOI:** 10.3390/foods15183251

**Published:** 2026-09-15

**Authors:** Esra Mesci Dindar, Dılhun Keriman Arserim-Uçar, Adnan Ayna

**Affiliations:** 1 Department of Medical Services and Techniques, Şiran Mustafa Beyaz Vocational High School, Gümüşhane University, Gümüşhane 29100, Türkiye; esra.mesci@gumushane.edu.tr; 2 Department of Nutrition and Dietetics, Faculty of Health Sciences, Bingöl University, Bingöl 12000, Türkiye; 3 Department of Chemistry, Faculty of Arts and Sciences, Bingöl University, Bingöl 12000, Türkiye

**Keywords:** propolis, colloidal delivery system, chitosan, phenolic encapsulation, functional food, phenolic compounds

## Abstract

In this study, a chitosan-based colloidal delivery systems (CDs) was developed as a natural delivery system for phenolic compounds from propolis extracts obtained via accelerated solvent extraction (ASE) and evaluated for improving the functional properties of walnut sausages. ASE propolis extract was incorporated into a chitosan (C)-based colloidal delivery system formulated with varying concentrations of diacetyl tartaric acid ester of mono- and diglycerides (DATEM) and lecithin (L). The formulation containing 2.5% chitosan, 12% DATEM and propolis, with a particle size of 265.9 nm and a ζ-potential of +40.8 mV, was selected for further characterization and food applications. The phenolic encapsulation of CDs depended on the structural characteristics of individual phenolic compounds, with chrysin (70.51%), vanillin (65.12%), and benzoic acid (51.26%) exhibiting the highest encapsulation; CD-treated walnut sausages decreased the lipid oxidation and improved the storage stability of the walnut sausages during storage. These findings indicate that the chitosan-based colloidal delivery system is a promising carrier for improving the encapsulation of propolis phenolics in walnut sausages.

## 1. Introduction

Recently, consumers have become more aware of the relationship between nutrition and health, resulting in the development of functional foods, which contain naturally occurring bioactive compounds. The term functional foods refers to food that offers more than nutrition, since these foods can also promote health and prevent disease [1]. Therefore, the use of delivery systems to enhance the functionality of conventional foods through the incorporation of bioactive compounds is an innovative way of doing it without compromising their natural characteristics.

Propolis is a resinous substance collected by honeybees from plant exudates and is recognized for its complex chemical composition, particularly its richness in phenolic acids, flavonoids, and aromatic compounds [2]. Several studies have demonstrated that propolis exhibits a wide range of biological activities, including antioxidant [3], antimicrobial [4,5], anti-inflammation [6], and anticancer properties [7], which emphasize its functional value in foods [8]. However, despite these beneficial properties, its application in food systems is limited due to its low water solubility, poor bioavailability, chemical instability, and undesirable taste, all of which may negatively affect consumer acceptance [9]. Therefore, there is a need to develop effective delivery strategies to improve the incorporation, stability, and bioaccessibility of propolis bioactive components in food applications.

To overcome these limitations, encapsulation technologies have been increasingly employed in the food sector as protective means and to act as delivery systems capable of enhancing stability, masking undesirable flavors, and enabling the controlled release of bioactive compounds [10]. To meet these requirements, a variety of encapsulation methods have been developed over time, including spray drying, spray cooling, extrusion, molecular inclusion, coacervation, ionic gelation, electrohydrodynamic methods, liposomal systems and emulsion-based approaches [11,12,13,14]. Among these, biopolymer-based colloidal delivery systems stand out for effectively incorporating bioactive compounds into food matrices and shielding them from environmental conditions [15]. The physicochemical properties of these systems are modulated by non-covalent interactions, such as electrostatic forces, hydrogen bonding, and hydrophobic interactions among biopolymers, surfactants, and bioactive compounds [16,17]. Among polysaccharide-based carriers, chitosan stands out as a particularly promising functional biopolymer for delivering natural bioactive compounds in food systems [18]. Derived from chitin through deacetylation, chitosan has been widely explored because it is biocompatible, biodegradable, non-toxic, and intrinsically antimicrobial [19]. Its cationic character enables strong electrostatic and other noncovalent interactions with bioactive molecules, making it suitable for constructing stable colloidal carrier systems, including drug delivery, Pickering emulsions, hydrogels, micro- and nanoparticles, and edible films [20,21,22,23]. Chitosan-based delivery systems can protect sensitive natural compounds from degradation while improving their bioavailability, thereby enhancing their overall effectiveness in food applications [24]. Consequently, these newly engineered delivery systems represent a promising strategy to enhance the bioavailability and stability of natural bioactive compounds, including essential oils [25], natural extracts [26], and honeybee products [27] for food applications.

A variety of chitosan-based delivery systems have been documented for the encapsulation of propolis extracts, including nanoparticles [28], nanogels [29], coacervates [30], and emulsions [31]. Collectively, these studies demonstrated the potential of chitosan-based carriers to enhance the delivery and protection of bioactive compounds in food applications. Additionally, propolis has also been incorporated into various food matrices such as meat, dairy products and yogurt, in both free and/or encapsulated forms to utilize its functional properties [32,33].

Despite these advances, limited information is available on the encapsulation and food applications of propolis extracts obtained via accelerated solvent extraction (ASE) when incorporated into chitosan-based colloidal carrier systems. Addressing this knowledge gap is essential to developing effective natural delivery systems that improve the stability of propolis phenolics and food applications. For that reason, the present study aimed to develop a chitosan-based colloidal delivery system (CD) for encapsulating propolis phenolics. This study hypothesized that a chitosan-based colloidal delivery system containing ASE propolis extract, stabilized by surfactant–biopolymer interactions, would effectively encapsulate and preserve the bioactive constituents and antioxidant capacity of popolis phenolics. Additionally, the incorporation of this propolis-loaded delivery system into walnut formulations is expected to enhance oxidative stability during storage.

## 2. Materials and Methods

### 2.1. Chemicals

Raw propolis was collected from the Bingöl Beekeepers Association (Bingöl, Türkiye) and stored at −20 °C until further processing. Chitosan (low molecular weight, degree of deacetylation 75–85%) was obtained from Sigma Aldrich (Darmstadt, Germany). Diacetyl tartaric acid ester of mono- and diglycerides (DATEM) and lecithin were kindly provided by DuPont (Brabrand, Denmark) and Cargill (Hamburg, Germany), respectively. Starch and carob molasses were purchased from a local market in Bingöl, Türkiye. All solvents used for chromatographic analyses were LC–MS grade (Sigma-Aldrich, Germany).

### 2.2. Preparation of Propolis Extract

The preparation of the propolis extract follows the procedures proposed by [34] with some modifications. Briefly, 0.5 g of raw propolis (P) was placed in an 11 mL stainless steel extraction cell and subjected to accelerated solvent extraction (ASE) system (Thermo Dionex ASE-350, Waltham, MA, USA). The extraction was performed using 70% (*v*/*v*) ethanol under the following settings: temperature of 60 °C, pressure of 1600 psi, flush volume of 80%, purge time of 60 s, static extraction time of 5 min, and two extraction cycles. The obtained extract was subsequently concentrated using a rotary evaporator (BUCHI/R300, Flawil, Switzerland) at 60 °C, lyophilized and stored at −20 °C until analysis.

### 2.3. Synthesis of Propolis-Loaded Chitosan-Based Colloidal Delivery Systems

Propolis-loaded chitosan-based colloidal delivery systems (CDs) were prepared according to previously reported methods [35,36], following a step-by-step sequence detailed below.

(1) Chitosan solutions at 2.5% (*w*/*v*) and 5% (*w*/*v*) were prepared in 30% (*v*/*v*) glacial acetic acid. (2) Subsequently, ethanol (2% *v*/*v*), propolis (2% *w*/*w*; by weight of chitosan), and (3) surfactant, DATEM or lecithin (0–12% *v*/*v*) relative to the volume of the chitosan solution, were added to the formulation. (4) The pH of all systems was adjusted to 4.8 using 0.1 N NaOH and/or 0.1 N HCl. Ultrasonication was performed for 30 s at 50% amplitude using a probe sonicator (Bandelin, Berlin, Germany) at each preparation step. The system was formulated as a chitosan-based colloidal delivery system. To develop the formulation, two main variables were evaluated: (i) chitosan concentration (2.5% or 5% *w*/*v*) and (ii) surfactant type and concentration (DATEM or lecithin, 0–12% *v*/*v*). Both propolis-loaded (P + CD) and propolis-free (P − CD) colloidal systems were prepared for comparative analysis. Particle size distribution and ζ-potential analyses were conducted to assess system stability as a function of the colloidal formulation parameters.

### 2.4. Particle Size, ζ-Potential and Polydispersity Index Determination

The particle size, ζ-potential, and polydispersity index (PDI) of the resulting CD formulations were measured by dynamic light scattering (DLS) on a Zetasizer Nano system (Malvern Instruments Ltd., Malvern, UK) at 25 °C. To minimize multiple scattering, the CD formulations were diluted tenfold with ultrapure water (1:10, *v*/*v*) before analysis. All measurements were performed in triplicate.

### 2.5. Fourier Transform Infrared (FTIR) Spectrum

FT-IR spectrum of individual components (chitosan, DATEM, lecithin, and propolis) and of the colloidal systems were recorded on an ATR-FT-IR spectrometer (Perkin-Elmer Spectrum 100A, Shelton, CT, USA). Spectra were collected over the wavenumber range of 400–4000 cm^−1^ at a resolution of 4 cm^−1^, with 64 scans per sample.

### 2.6. Confocal Laser Scanning Microscopy (CLSM)

CLSM was performed according to the published protocol [37] with slight modifications for the present study. The microstructure of the P + CD and P − CD systems was investigated using a Zeiss LSM 880 confocal laser scanning microscope with Airyscan technology (Zeiss, Jena, Germany). The microscope was equipped with a 63× oil-immersion lens (numerical aperture 1.4), a photomultiplier tube, and a sensitive GaAsP detector. The Zeiss Application Suite ZEN software was used to analyze images. Nile Red (10 µL of 0.1% *w*/*v* in DMSO) and Calcofluor White (100 µL of 1% *w*/*v* in Milli-Q water) were added to 0.5 mL of the P + CD and P − CD colloidal systems. Stained samples were mounted onto glass coverslips and illuminated at excitation wavelengths of 538 nm for Nile Red staining and 335 nm for Calcofluor White staining [38].

### 2.7. Thermogravimetric Analysis

The thermostability of chitosan, DATEM, lecithin, propolis, P + CD, and P − CD was analyzed using a Simultaneous Thermal Analyzer (STA 600, PerkinElmer, Shelton, CT, USA). Samples were heated from 30 to 700 °C at the rate of 10 °C min^−1^ in a nitrogen gas environment, with the mass loss curve being tracked continuously [14].

### 2.8. Phenolic Profile of the Propolis ASE Extract and CDs

The phenolic composition of the propolis extract and P + CD was determined using Ultra-High-Performance Liquid Chromatography (UHPLC) coupled to Orbitrap High-Resolution Mass Spectrometry (HRMS). The separation was performed on a Dionex Ultimate 3000 RS UHPLC system. The Orbitrap mass analyzer was calibrated in positive ion mode using the Pierce LTQ Velos ESI Positive Ion Calibration Solution and in negative ion mode using the Pierce Negative Ion Calibration Solution via an automated syringe pump (Thermo Fisher Scientific, Waltham, MA, USA). Chromatographic separations were performed on a Phenomenex Gemini NX-C18 column (100 × 2.0 mm i.d., 110 Å pore size, 3 μm particle size), held at 30 °C. The mobile phase solvent system was prepared using ultrapure water acidified with 2% *v*/*v* glacial acetic acid (A) and filtered methanol (B) suitable for LC–MS. The gradient started at 0–2 min with 100% A and 0% B, then increased linearly in B from 0% to 98% between 2 and 13 min. The mobile phase was maintained at 2% A and 98% B from 13 to 15.9 min, returned to 100% A and 0% B from 15.9 to 16 min, and was maintained at the initial conditions until 19 min. Samples were injected at 20 μL with a constant flow rate of 0.3 mL min^−1^. Mass spectrometric data were obtained by full-scan MS in both positive and negative ionization modes using all-ion fragmentation (AIF). The data was acquired using Xcalibur software (version 2.1.0.1140, Thermo Fisher Scientific), and data analysis was performed using TraceFinder software (version 3.2, Thermo Fisher Scientific) [39,40]. Mass spectrometric analysis was performed in positive and negative ionization modes using full-scan acquisition (m/z 60–800) at a resolution of 17,500 FWHM. Phenolic compounds were identified based on retention time, accurate parent ion (m/z), ionization mode, and fragment ions obtained from all-ion fragmentation (AIF) (Supplementary Materials, Table S1).

### 2.9. Phenolic Encapsulation Efficiency (EE %)

The phenolic encapsulation efficiency (EE %) was determined following a modified procedure based on the method of [35]. The quantity of encapsulated polyphenols from the chitosan-based delivery system (CDs) was determined via UHPLC-Orbitrap-HRMS, and the encapsulation efficiency was calculated using the following equation:

(1) $$EE\;(\%)=\frac{Total\;amount\;of\;phenolic\;compound\;in\;CDs}{Total\;amount\;of\;phenolic\;compound\;in\;propolis\;extract}\times 100$$

### 2.10. Walnut Sausages Production with CDs

Walnut sausage (WS) preparation were developed according to the procedure described in [41] with some modifications, incorporating propolis-loaded chitosan-based colloidal delivery systems (CDs) to the formulation. The CD formulations used contained 2.5% chitosan, 12% DATEM, and propolis (2% *w*/*w*; by weight of chitosan). The acetic acid present in the CDs was first evaporated. Carob molasses (10 mL) was combined with starch (2% *w*/*v*, based on the volume of carob molasses) and heated to 66 °C with continuous stirring until the mixture reached the desired consistency. After cooling to 40 °C, varying volumes of CD were added to generate three propolis-enriched formulations: −WS + 10CD (2% *w*/*w* propolis), WS + 20CD (4% *w*/*w* propolis), and WS + 40CD (8% *w*/*w* propolis), with propolis concentrations calculated relative to the final walnut sausage formulation weight. A control formulation without CD was similarly prepared WS (Control). Walnut sausages were subsequently coated by sequential immersion (three cycles, 15 s per cycle) in the respective formulations. The coated walnut sausages were dried at 40 °C for 3 days, vacuum-packaged, and stored at room temperature (22 ± 2 °C) in until analysis.

### 2.11. Thiobarbituric Acid Reactive Substances (TBARS) Assay

Lipid peroxide content in walnut sausage samples was assessed via TBARS assay according to previously published method [42] with modifications. Food samples (1 g) were homogenized in 20 mL of 0.5% (*w*/*v*) thiobarbituric acid prepared in 20% (*w*/*v*) trichloroacetic acid. The resulting homogenate was heated at 85 °C for 90 min, and rapidly cooled in an ice bath and centrifuged at 10,000× *g* for 10 min. The absorbance of the supernatant was measured spectrophotometrically at 532 nm Malondialdehyde (MDA) concentration was determined using a calibration curve prepared with 1,1,3,3-tetraethoxypropane (TEP) as the standard reference. TBARS values were expressed as micrograms of MDA equivalents per gram of sample (µg MDA eq g^−1^).

### 2.12. Preparation of Food Extract

Food samples (2 g) were extracted using 5 mL of a solvent system comprising 75% methanol, 0.1% formic acid and water (*v*/*v*/*v*). The mixture was then centrifuged at 2700 rpm for 10 min at 4 °C. The supernatant was collected, and the remaining precipitate was subjected to two sequential extractions using the same procedure [43]. The supernatants were used to determine the total phenolic content and antioxidant capacity.

### 2.13. The Total Phenolic Content Analysis

Total phenolic content (TPC) in food extracts was determined using the Folin–Ciocalteu assay. Food extracts were reacted with 0.2 N Folin–Ciocalteu reagent and 7.5% sodium carbonate. The reaction mixture was incubated in the dark at room temperature for 120 min Absorbance was measured spectrophotometrically at 765 nm using a UV–VIS spectrophotometer. The results were presented as mg GAE per g food [44].

### 2.14. CUPRAC (Cupric Ion Reducing Antioxidant Capacity) Assay

The CUPRAC assay was performed according to a previously published method [45] with some modifications. Briefly, 100 µL of samples were mixed with 1 mL of each solution of 10^−2^ M copper (II) chloride dihydrate (CuCl_2_·2H_2_O), 7.5 × 10^−3^ M neocuproine, 1 M ammonium acetate buffer, and distilled water. After incubation for 30 min, absorbance was measured at 450 nm. The results were expressed as Trolox equivalents [45].

### 2.15. Statistics

Statistical analyses were conducted using Origin 2025b software. For colloidal system studies, one-way analysis of variance (ANOVA) was performed, followed by Tukey’s multiple comparison test, with statistical significance set at *p* < 0.05. Normality was evaluated using the Shapiro–Wilk test. For walnut sausage samples, the effects of food groups and storage time were evaluated using two-way repeated-measures ANOVA, also followed by Tukey’s multiple comparison test, with a significance level of *p* < 0.05. All experiments were conducted in triplicate, and data are presented as mean ± standard deviation (n = 3).

## 3. Results and Discussion

### 3.1. Particle Size, ζ-Potential and Polydispersity Index Analysis

The characteristics of colloidal systems prepared with chitosan (2.5% C and 5% C), DATEM (D), or lecithin (L) at different concentrations (0–12% *v*/*v*) are presented in Table 1, Table 2, Table 3 and Table 4. Most formulations exhibited positive ζ-potential values exceeding +30 mV. The ζ-potential of the droplets became rather stable to coalescence and positive in both the 2.5% C and 5% C chitosan formulations, indicating that the droplets were saturated with chitosan [46].

Some formulations containing 5% (*w*/*v*) chitosan generally exhibited slightly higher ζ-potential values than those prepared with 2.5% (*w*/*v*) chitosan, suggesting that increasing the chitosan concentration enhanced the surface charge density of the dispersed particles. Due to the considerable electrostatic repulsion among the highly positively charged droplets, it is feasible to hypothesize that the emulsions would remain stable against droplet aggregation at sufficiently high chitosan concentrations. It is probable that the droplets may be able to adsorb to the surfaces of several droplets [47].

The incorporation of propolis further increased ζ-potential, particularly in the DATEM-stabilized formulations, implying favorable interactions between propolis constituents and the chitosan matrix that promoted greater surface charge. An exception was observed in 5% C (chitosan) + 6% DATEM + P (propolis) formulation, which exhibited a marked decrease in ζ-potential. This behavior may be attributed to partial charge neutralization resulting from stronger interactions between the propolis constituents and DATEM at this specific composition.

Particle size analysis revealed that all formulations were within the submicron ranging from approximately 200 to 700 nm. Colloidal systems prepared with 2.5% exhibited smaller particle sizes compared to 5% C formulations, indicating that polymer concentration promoted the formation of larger particles likely due to enhanced viscosity; non-adsorption or excess chitosan may cause particle aggregation and bridging flocculation effects [36,46,47].

In DATEM-containing formulations, increasing the emulsifier concentration from 0% to 12% generally led to changes in particle size, suggesting that interfacial coverage and the formation of more compact particles were involved [48]. When all propolis-loaded formulations were evaluated at both chitosan concentrations (2.5% and 5.0% *w*/*v*), the formulation containing 2.5% (*w*/*v*) chitosan and 12% DATEM with propolis was selected as the optimum formulation based on the combined evaluation of its highest zeta potential, and small particle size. In contrast, lecithin-based formulations showed greater variability in particle size, particularly at lower emulsifier concentrations, which may reflect complex interfacial behavior associated with the amphiphilic nature of lecithin [36]. These results collectively illustrate how the combined effects of chitosan concentration, the nature and amount of the emulsifier, and propolis ASE extract influence particle size and surface charge, and thus the colloidal properties of the resulting systems.

The physicochemical characteristics of the chitosan–propolis particulate systems observed in this study is consistent with previous reports on chitosan-based colloidal delivery systems. The increase in particle size with higher chitosan concentration (5% *w*/*v* vs. 2.5% *w*/*v*) may be attributed to stronger polymer chain interactions and higher viscosity [47]. Surfactant hydrophilic–lipophilic balance (HLB) affects droplet size, which in turn influences physical stability, and the PDI of the CDs indicates a monodisperse distribution [36].

Similar concentration-dependent increases in particle size have been reported for chitosan nanoparticles, where stronger intermolecular interactions and reduced diffusion efficiency resulted in larger particle diameters [49,50]. Most formulations exhibited positive ζ-potential values exceeding +30 mV, indicating strong electrostatic repulsion between particles and resistance to aggregation. This behavior is characteristic of chitosan-based colloidal systems and arises from the protonation of amino groups under acidic conditions, which generates a positively charged particle surface and promotes electrostatic repulsion between droplets, thereby reducing aggregation [51]. The incorporation of propolis further increased the ζ-potential values for several formulations, particularly those stabilized with DATEM, suggesting that interactions between propolis constituents and the chitosan matrix altered the particles surface characteristics. Similar effects have been reported for bioactive-loaded polysaccharide carriers, in which the physicochemical properties of the polymer composition, driving forces for delivery systems, and the chemical composition of encapsulated compounds influence the kinetic stability of the delivery system [36]. In contrast, the pronounced decrease in ζ-potential observed for the 5% C (chitosan) + 6% DATEM + P (propolis) formulation may indicate partial charge neutralization resulting from stronger interaction among chitosan, DATEM and propolis phenolics. Such surfactant–polymer interactions are known to modify interfacial organization and electrokinetic behavior in polysaccharide-based colloidal systems, thereby affecting overall colloidal stability [52].

### 3.2. FT-IR Measurements

**Figure 1:**
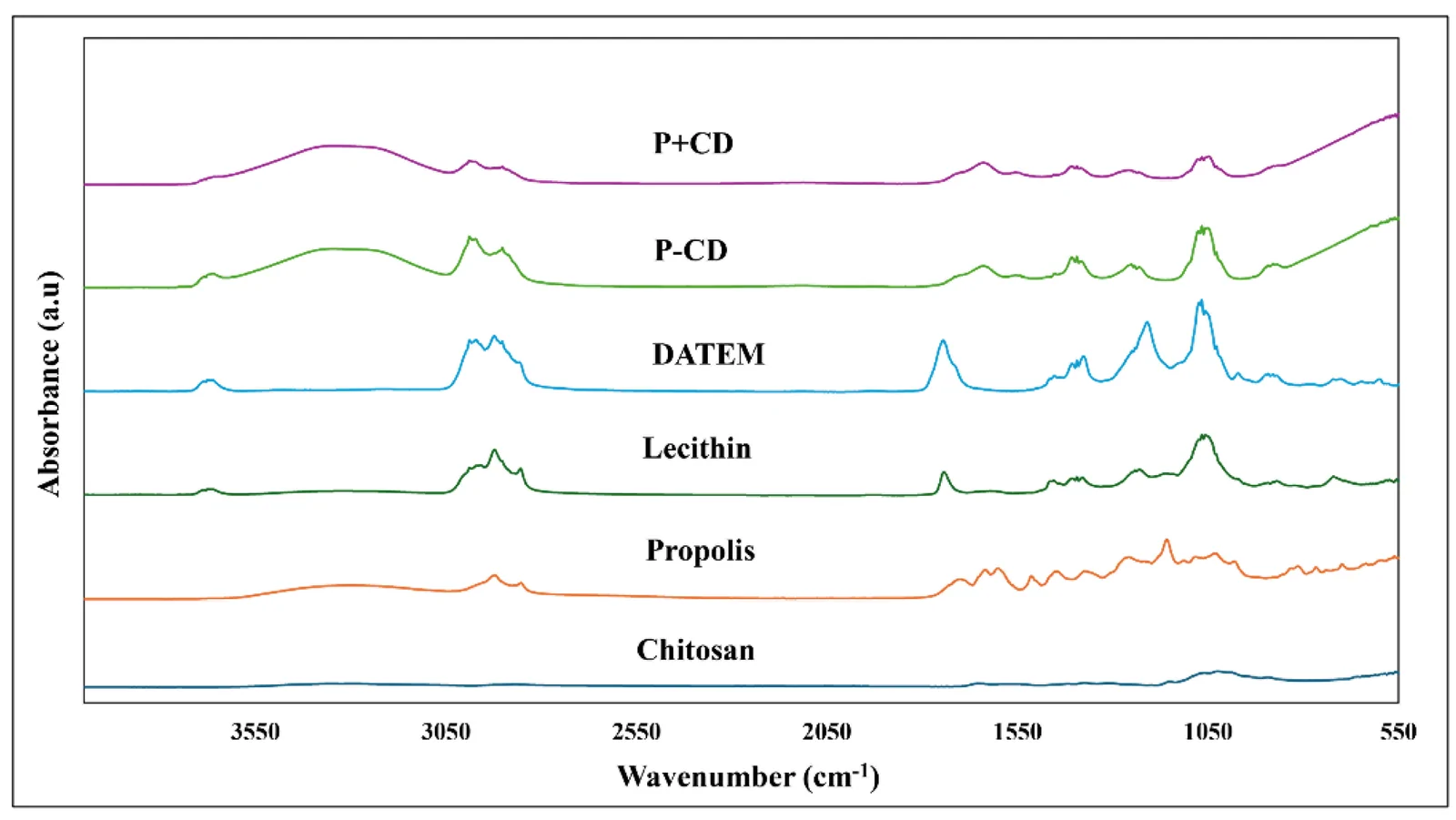
FT-IR spectrum of individual materials: propolis (P), chitosan (C), lecithin (L), and DATEM, and their respective colloidal systems with propolis (P + CD) and propolis-free (P − CD) in the 4000–400 cm^−1^ region.

The FTIR spectra provide evidence of molecular interactions between chitosan, DATEM, lecithin, and propolis in the developed colloidal systems (Figure 1). The spectrum for propolis (P) shows a broad peak at ~3300 cm^−1^, indicative of O–H and N-H stretching vibrations, in addition to characteristic C–H stretching absorptions at ~2922 and ~2852 cm^−1^ [53]. The FTIR spectra of the propolis extracts exhibit characteristic absorption bands at 1090–1020 cm^−1^ attributable to C–O stretching, at 1645–1635 cm^−1^ corresponding to C=O stretching, at 2980–2870 cm^−1^ assigned to C–H stretching, and at 3350–3250 cm^−1^ related to O-H stretching. The absorption bands in the region of 1634–1638 cm^−1^ can be assigned to C=O stretching, with contributions from flavonoids, lipids, and amino acids, as well as absorption at 1511 cm^−1^ for C-C stretching of aromatic rings. C=O stretching is also observed for aliphatic aldehydes and ketones [53].

Pure chitosan (C) has a broad absorption band around 3290 cm^−1^ because of the overlap of O–H and N–H bonding, thereby establishing its hydrogen-bonded polysaccharide network. The absorption band around 1652 cm^−1^ is ascribed to the amide I vibration (C=O stretching), along with a shoulder around 1374 cm^−1^ due to CH_3_ bending, as well as a band around 1030 cm^−1^ because of C–O stretching in the polysaccharide chain [54]. The absorption bands of DATEM and lecithin (L) are indicative of aliphatic C–H stretching vibrations around 2920–2850 cm^−1^, along with a high absorption in the 1200–1000 cm^−1^ range because of ester C–O bonds [48,55].

Compared with the spectra of the individual components, the colloidal formulations (P + CD and P − CD) exhibited slight band broadening and minor shifts in the O–H/N–H stretching region (approximately 3300 cm^−1^). These may be associated with hydrogen-bonding interactions between chitosan and constituents of propolis. Furthermore, variations in intensity as well as slight shifts in the region of 1650–1500 cm^−1^, attributable to C=O bonds, signify possible electrostatic binding or formation of a complex between protonated amino groups of chitosan and anionic constituents of either DATEM or lecithin [56].

### 3.3. Confocal Scanning Laser Microscopy

**Figure 2:**
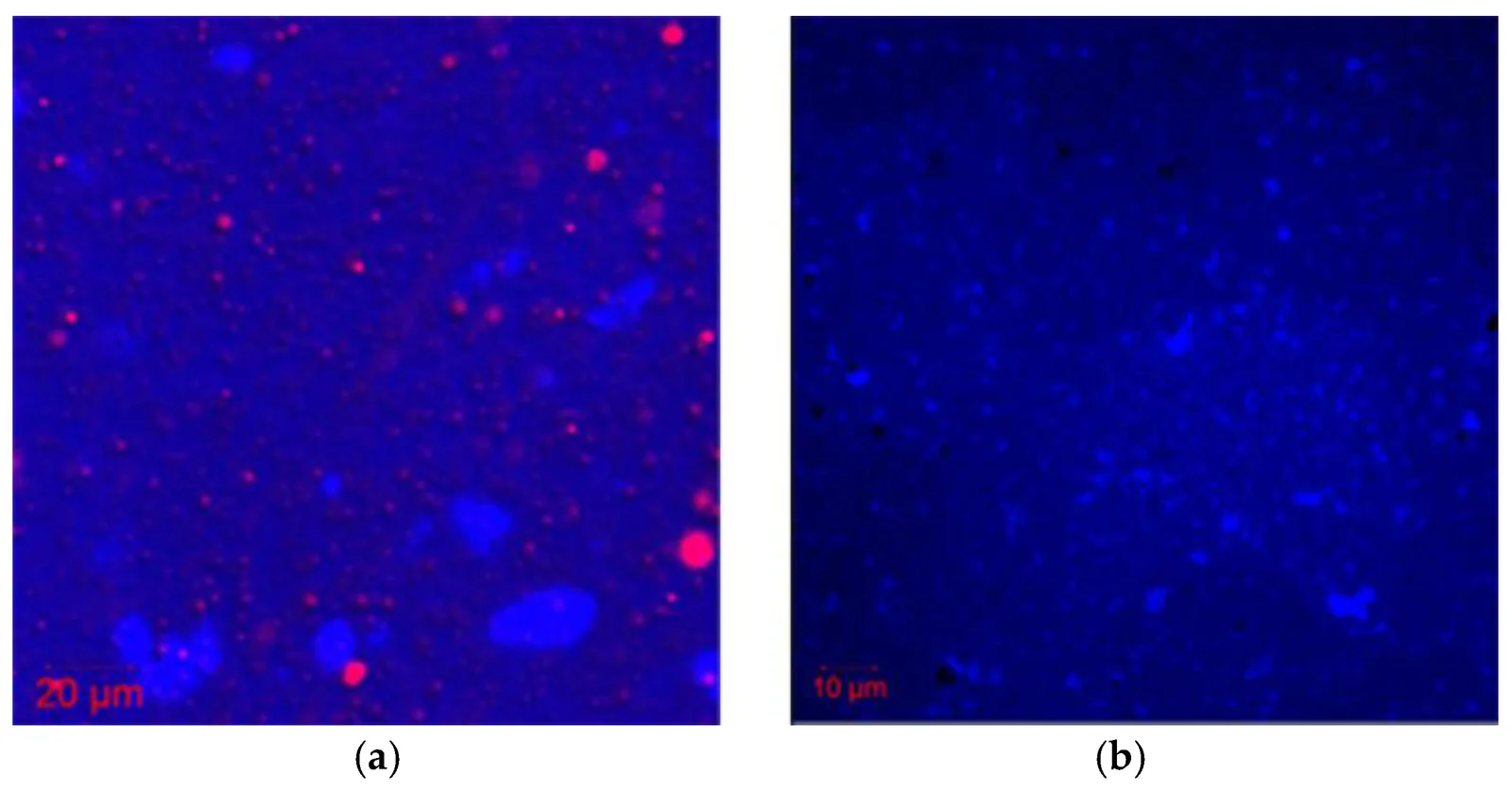
Microscopy images of the chitosan-based colloidal system: (**a**) P + CD (with propolis); (**b**) P − CD (propolis-free). Chitosan staining: blue; propolis staining: red. Scale bars: 20 μm for (**a**) and 10 μm for (**b**).

Confocal laser scanning microscopy (CLSM) provided morphological information on the microstructure of the chitosan-based colloidal delivery system (Figure 2). The fluorescence images revealed a homogeneous distribution of the propolis phase throughout the continuous chitosan matrix, with no significant phase separation. CLSM images indicated a spherical CD morphology [57].

### 3.4. Thermogravimetric Analysis of Chitosan-Based Particle Formulations and Individual Components

**Figure 3:**
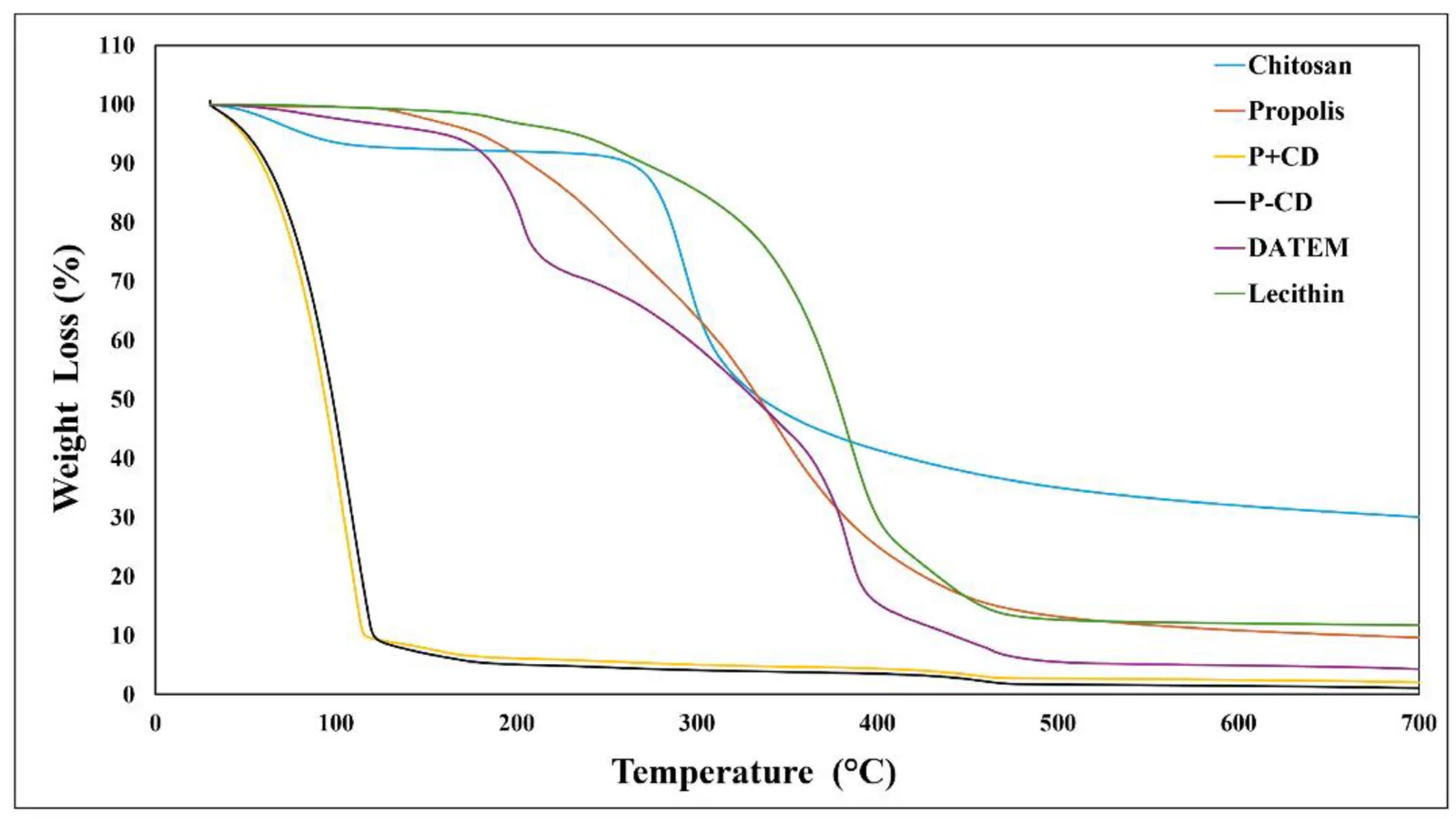
Thermogravimetric (TGA) curves of chitosan, DATEM, propolis, colloidal systems with propolis (P + CD) and without propolis (P − CD).

Thermogravimetric analysis (TGA) thermograms revealed the characteristic thermal decomposition profiles of chitosan, DATEM, lecithin, propolis, and the composite colloidal systems (Figure 3). Overall weight losses in the temperature range of 30–700 °C were 68.74% for chitosan-based systems, 93.6% and 88.35% for lecithin- and DATEM-containing formulations, respectively, 97.2% for the propolis-loaded formulation (P + CD), and 98.8% for the propolis-free formulation (P − CD). All chitosan-containing samples showed a multi-stage degradation profile consistent with recent research findings on the thermal properties of chitosan bioactive or polyphenol composites. The initial weight loss observed between 0 and 150 °C was attributed to the removal of physically adsorbed and hydrogen-bonded water molecules [58]. The main decomposition area, occurring between 150 and 600 °C, corresponded to the thermal decomposition of the polysaccharide backbone and incorporated bioactive constituents. In the P + CD formulation, compared with P − CD, a shift in the onset of major weight loss to approximately 167 °C suggests that propolis incorporation modifies the thermal decomposition behavior of the chitosan matrix, in agreement with previous reports on polysaccharide–polyphenol systems [59]. Likewise, propolis alone exhibited broad degradation profile over an expanded temperature range, reflecting the presence of diverse constituents such as polyphenols, waxes, and volatile compounds. DATEM showed a principal decomposition phase between ~212 and 390 °C, where the greatest weight loss was observed. Collectively, these findings indicate that both the addition of propolis and emulsifiers to the chitosan matrix caused dramatic changes in the thermal degradation process. Bonds between the propolis, chitosan, and DATEM formed by electrostatic interactions between oppositely charged groups exhibit greater susceptibility to dissociation and degradation under high temperatures [60].

### 3.5. Phenolic Compounds of the Propolis Extract and CD

The UHPLC-Orbitrap HRMS profiles of phenolic acids and flavonoids identified in the analyzed sample, both the qualitative and quantitative, are shown in Table 5. Among the phenolic acids, the cinnamic derivatives were predominant, with ferulic acid (2868.86 µg/g), caffeic acid (2111.16 µg/g), caffeic acid phenethyl ester (CAPE, 1426.94 µg/g), and p-coumaric acid (1139.64 µg/g) representing the major constituents, whereas chlorogenic acid was detected only at trace levels. Flavonoids were the most abundant class, with high levels of naringenin (3874.19 µg/g), galangin (3369.42 µg/g), and chrysin (2641.85 µg/g). The profile also included both aglycones and glycosylated flavonoids (e.g., rutin, isoquercitrin, hyperoside, astragalin, and narcissin), alongside minor anthocyanins (cyanidin 3-glucoside). Overall, the combined dominance of cinnamic acid derivatives and flavonoids—together with CAPE, chrysin, galangin, and naringenin—indicates a polyphenol-rich sample with substantial potential biological relevance.

The phenolic acid composition of propolis is highly variable and depends on the botanical source, geographical origin, and seasonal conditions, resulting in unique chemical profiles for each propolis type. The phenolic composition of the Bulgaria type is particularly characterized by pinobanksin-3-O-propionate, chrysin, pinocembrin, caffeic acid benzyl ester, p-coumaric acid, and caffeic acid [61]. UHPLC-Orbitrap HRMS analysis revealed that the phenolic profile of the propolis-loaded colloidal system (2.5% chitosan, 12% DATEM, and propolis) was rich and structurally diverse containing phenolic acids and multiple subcategories of flavonoids (Table 6). Among phenolic acids, cinnamic acid derivatives were with ferulic acid (1035.55 µg/g), coumaric acid (436.17 µg/g), caffeic acid (394.32 µg/g), and caffeic acid phenethyl ester (384.80 µg/g) the major constituents indicating a strong presence of redox active compounds. Benzoic acid derivatives, such as benzoic acid (509.41 µg/g) and vanillin (282.93 µg/g) were also detected in considerable amounts. Flavonoids constituted the major phytochemical group within the system with high levels of chrysin (1862.82 µg/g) and galangin (1082.76 µg/g), reflecting the abundance of bioactive aglycone flavonoids. Additional flavonoids such as naringenin (723.11 µg/g), apigenin (205.63 µg/g), acacetin (241.24 µg/g), and luteolin (43.25 µg/g) were also identified. In contrast, glycosylated flavonoids, including rutin, apigenin-7-O-glucoside, hyperoside, isoquercitrin, and narcissin, were present at relatively lower levels, suggesting limited encapsulation of more polar derivatives within the system. Thus, the presence of flavonoids and cinnamic acids indicates effective complexation within the biopolymer–surfactant interaction, contributing to the stabilization and bioactivity of the colloidal delivery system.

### 3.6. Phenolic Encapsulation

The phenolic encapsulation of the developed propolis-loaded colloidal system (2.5% chitosan, 12% DATEM, and propolis) was evaluated to assess the potential effectiveness of the chitosan-based colloidal matrix as a carrier for propolis ASE extract phenolic compounds (Table 7). Phenolic encapsulation efficiency (EE) was calculated for each compound using Equation (1). The results demonstrated that encapsulation efficiency was significantly influenced by the chemical structure of individual phenolic compounds, highlighting the critical role of molecular characteristics in determining the effectiveness of the encapsulation process. Among phenolic acids, high encapsulation efficiency was observed for vanillin (65.12 ± 0.62%) and benzoic acid (51.26 ± 0.80%), while the lowest was recorded for caffeic acid (18.83 ± 2.47%). Ferulic acid, coumaric acid, chlorogenic acid, and CAPE recorded moderate encapsulation efficiency, estimated at 36.10 ± 0.90%, 38.51 ± 3.93%, 38.69 ± 16.78%, and 27.24 ± 3.96%, respectively. Aglycone flavones such as chrysin showed the highest encapsulation efficiency of 70.51 ± 0.25%, indicating strong interactions with the chitosan colloidal matrix. The higher encapsulation efficiency of chrysin may be associated with its relatively hydrophobic structure and its potential non-covalent interactions with chitosan, surfactants, and other system components [62]. In contrast, more polar phenolics showed lower encapsulation because of their solubility characteristics and interactions with the components of the colloidal system. Moderate encapsulation efficiency was observed in apigenin, galangin, luteolin, and acacetin at 38.47 ± 0.33%, 32.29 ± 3.39%, 28.09 ± 3.56%, and 27.34 ± 0.58%, respectively. As opposed to that, the corresponding flavonoid glycosides, such as rutin, narcissin, nicotiflorin, and astragalin, showed apparently low phenolic encapsulation efficiency of 8.29 ± 0.53%, 9.53 ± 1.29%, 17.35 ± 1.83%, and 16.24 ± 2.02%, respectively, likely due to high hydrophilicity and reduced interfacial activity [63].

### 3.7. TBARS Values of Walnut Sausage Formulated with CDs During Storage

**Figure 4:**
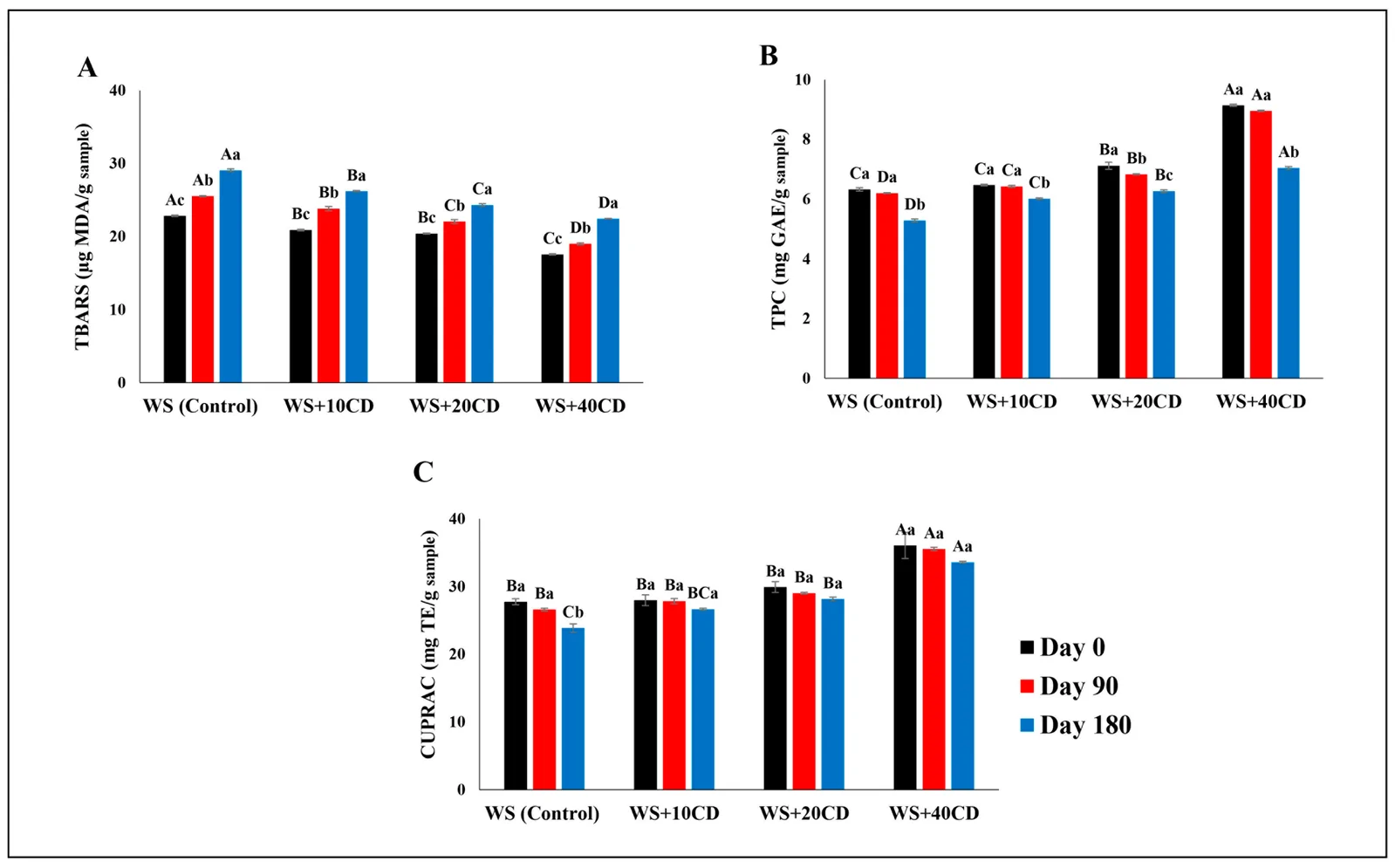
Changes in (**A**) TBARS, (**B**) total phenolic content (TPC), and (**C**) CUPRAC antioxidant activity values of walnut sausages with and without CD samples during 180 days of storage. Different capital letters (A–D) indicate statistically significant differences among WS samples (*p* < 0.05). Different lowercase letters (a–c) indicate statistically significant differences among storage days (*p* < 0.05). All values are expressed as means ± SD (n = 3). WS: control walnut sausage, WS + 10CD, WS + 20CD, and WS + 40CD are chitosan-based colloidal delivery system formulations, respectively.

Lipid oxidation, assessed based on TBARS values, in all walnut sausage (WS) samples during storage from day 0 to day 180 is presented in Figure 4A. The control (WS) group exhibited the highest TBARS values throughout the storage period, increasing from 22.84 µg MDA/g on day 0 to 29.06 µg MDA/g on day 180, indicating progressive lipid oxidation in the absence of the incorporated antioxidant formulation. In contrast, all treated groups exhibited significantly lower TBARS values than the control at all storage times, demonstrating the inhibitory effect of the incorporated colloidal systems on lipid peroxidation. A concentration-dependent effect was also observed, as increasing concentrations of the colloidal system (10CD, 20CD, and 40CD) resulted in progressively lower TBARS values. Among the treated groups, WS + 40CD consistently showed the lowest TBARS values, indicating that a higher incorporation level of the colloidal system provided greater protection against oxidative deterioration during storage.

Walnuts are particularly susceptible to lipid oxidation due to their high polyunsaturated fatty acid (PUFA) content and sensitivity to environmental factors including heat, light, oxygen, and moisture [64]. Lipid peroxidation in walnuts can be effectively reduced by antioxidant rich formulations containing phenolic compounds [65]. Propolis, which is characterized by a high content of bioactive antioxidant compounds, has been successfully incorporated into various meat products, including beef patties [66], and frozen lamb burgers [67], resulting in reduced lipid peroxidation levels. The present TBARS findings demonstrate that incorporation of the propolis-containing colloidal delivery system significantly prevented lipid peroxidation in walnut sausage samples during storage, evidencing that phenolic compounds derived from propolis ASE extract effectively scavenged free radicals, thereby decreasing the lipid oxidation and improving the storage stability of the walnut sausage. Moreover, the progressively lower TBARS values observed with increasing colloidal system concentrations suggest a concentration-dependent protective effect against oxidative deterioration.

### 3.8. Total Phenolic Content and Antioxidant Capacity of Walnut Sausages Formulated with CDs During Storage

TPC values for CDs formulations are shown in Figure 4B. On day 0, the WS group exhibited the lowest TPC value (6.33 mg GAE/g sample), whereas all colloidal systems (CDs)-treated groups showed higher TPC values, confirming the successful encapsulation of phenolic compounds into the developed formulations. During storage, a reduction in TPC was observed across all samples, which may be attributed to degradation reactions, oxidation, and interactions between phenolic compounds and the food matrix. The extent of this reduction was formulation-dependent. The WS group showed a more pronounced decrease, reaching 5.29 mg GAE/g sample on day 180, whereas all colloidal system samples maintained higher TPC levels throughout the storage period. Among the treated formulations, increasing colloidal concentration improved the encapsulation of phenolic compounds. In particular, WS + 40CD consistently exhibited the highest TPC values during storage, indicating higher encapsulation of propolis phenolic compounds.

The antioxidant activity of the walnut sausage samples is presented in Figure 4C. On day 0, the WS group exhibited the lowest antioxidant activity (27.73 mg TE/g sample), whereas all CD-treated groups showed significantly higher values (*p* < 0.05), indicating the successful incorporation and encapsulation of antioxidant compounds within the samples. A dose-dependent antioxidant activity was observed. During storage, there was a gradual loss of antioxidant activity across all groups, attributed to possible oxidation and/or interactions between antioxidants and food matrix components. The antioxidant activity of the control group and the walnut sausage with CDs formulation decreased during storage.

Walnuts exhibit substantial antioxidant potential, which is primarily attributed to their diverse and abundant phenolic constituents, including phenolic acids, flavonoids, and quinone derivatives [68]. Likewise, carob fruit is characterized by a complex phenolic profile comprising hydroxybenzoic acids, flavonoids, and flavan-3-ols. Among the principal phenolic compounds identified in carob are gallic acid, syringic acid, p-coumaric acid, rutin, myricetin, catechin, and epicatechin, which collectively contribute to its pronounced antioxidant capacity [69]. In the present formulation, the combination of propolis, walnuts, and carob increased TPC and antioxidant activity, likely due to the cumulative effect of their phenolic profiles.

## 4. Conclusions

In this research, an innovative encapsulating technique for the delivery of ASE propolis extract phenolics was established utilizing chitosan-based colloidal systems enhanced with lecithin and DATEM. The formulation containing 2.5% chitosan, 12% DATEM, and propolis, with a particle size of 265.9 nm and a ζ-potential of +40.8 mV, in walnut sausage applications prevents lipid oxidation and improves the storage stability of the walnut sausages. Chitosan-based colloidal systems (2.5% chitosan, 12% DATEM) with propolis recovered the high amount of propolis phenolics. The limitations of this study are the absence of sensory evaluation, gastrointestinal bioaccessibility assessment, and microbial analysis, and physical stability of the colloidal system. Addressing these limitations in future studies will enable a more comprehensive assessment of the performance, stability, and application potential of the developed system. Chitosan-based delivery systems incorporating cost-effective, food-grade surfactants may represent promising platforms for the encapsulation and controlled delivery of bioactive compounds, particularly for food and nutritional applications.

## Figures and Tables

**Table 1:** Particle size, ζ-potential and PDI of propolis-containing (P + CD) 2.5% (*w*/*v*) chitosan formulations.

Chitosan (*w*/*v*)	DATEM (*v*/*v*)	ζ-Potential (mV)	Particle Size (nm)	PDI	Lecithin (*v*/*v*)	ζ-Potential (mV)	Particle Size (nm)	PDI
**2.5**	0	34.10 ± 7.69 ^a^	413.67± 2.55 ^a^	0.18 ± 0.02 ^a^	0	26.60± 0.90 ^b^	515.83± 5.27 ^a^	0.26 ± 0.01 ^a^
	2	34.63 ± 2.98 ^a^	343.33± 29.92 ^abc^	0.19 ± 0.03 ^a^	2	33.47± 1.98 ^ab^	334.90± 4.68 ^c^	0.14 ± 0.02 ^b^
	4	37.27 ± 3.42 ^a^	296.90± 25.65 ^abc^	0.16 ± 0.0 ^a^	4	34.83± 3.51 ^a^	341.77± 14.18 ^c^	0.16 ± 0.01 ^b^
	6	36.60 ± 3.04 ^a^	356.63± 6.41 ^b^	0.12 ± 0.09 ^a^	6	36.57± 2.87 ^a^	275.17± 20.16 ^d^	0.17 ± 0.01 ^b^
	8	34.57 ± 2.57 ^a^	280.30 ± 31.81 ^abc^	0.17 ± 0.02 ^a^	8	34.17± 2.25 ^a^	314.90± 14.75 ^c^	0.18 ± 0.03 ^ab^
	10	36.07 ± 3.40 ^a^	318.10 ± 11.88 ^bc^	0.23 ± 0.05 ^a^	10	36.73± 3.29 ^a^	390.20± 2.57 ^b^	0.17 ± 0.01 ^b^
	12	40.77 ± 1.85 ^a^	265.93 ± 2.44 ^c^	0.16 ± 0.04 ^a^	12	35.47± 2.31 ^a^	337.60± 0.70 ^c^	0.20 ± 0.04 ^ab^

All values are means ± SD, n = 3. Different lowercase letters (a–d) in the same column indicate statistically significant differences among system formulations (*p* < 0.05).

**Table 2:** Particle size, ζ-potential, and PDI of propolis-containing (P + CD) 5% (*w*/*v*) chitosan formulations.

Chitosan (*w*/*v*)	DATEM (*v*/*v*)	ζ-Potential (mV)	Particle Size (nm)	PDI	Lecithin (*v*/*v*)	ζ-Potential (mV)	Particle Size (nm)	PDI
**5**	0	37.23 ± 2.45 ^a^	456.07 ± 30.17 ^b^	0.19 ± 0.01 ^b^	0	26.70 ± 0.52 ^c^	707.33 ± 32.79 ^a^	0.23 ± 0.01 ^a^
	2	37.97 ± 4.31 ^a^	312.90 ± 34.14 ^cd^	0.20 ± 0.04 ^ab^	2	26.70 ± 0.61 ^c^	575.50 ± 8.26 ^a^	0.21 ± 0.02 ^a^
	4	36.50 ± 3.30 ^a^	399.60 ± 44.88 ^bc^	0.15 ± 0.01 ^b^	4	33.47 ± 2.90 ^b^	358.87 ± 18.07 ^c^	0.22 ± 0.01 ^a^
	6	13.73 ± 0.75 ^b^	564.07 ± 42.10 ^a^	0.37 ± 0.01 ^a^	6	40.47 ± 1.17 ^a^	566.40 ± 6.25 ^a^	0.32 ± 0.05 ^a^
	8	35.90 ± 2.71 ^a^	418.63 ± 5.04 ^b^	0.16 ± 0.01 ^b^	8	35.43 ± 2.39 ^ab^	401.20 ± 6.32	0.19 ± 0.02 ^a^
	10	36.50 ± 3.08 ^a^	302.10 ± 22.37 ^d^	0.18 ± 0.01 ^b^	10	36.17 ± 3.20 ^ab^	465.67 ± 11.50 ^b^	0.20 ± 0.04 ^a^
	12	36.17 ± 2.90 ^a^	307.40 ± 23.54 ^d^	0.16 ± 0.04 ^b^	12	36.47 ± 2.30 ^ab^	451.27 ± 6.95 ^b^	0.21 ± 0.01 ^a^

All values are means ± SD, n = 3. Different lowercase letters (a–d) in the same column indicate statistically significant differences among system formulations (*p* < 0.05).

**Table 3:** Particle size, ζ-potential, and PDI of chitosan formulations propolis-free (P − CD) at 2.5% (*w*/*v*).

Chitosan (*w*/*v*)	DATEM (*v*/*v*)	ζ-Potential (mV)	Particle Size (nm)	PDI	Lecithin (*v*/*v*)	ζ-Potential (mV)	Particle Size (nm)	PDI
**2.5**	0	38.67 ± 4.06 ^a^	489.47 ± 33.48 ^a^	0.61 ± 0.10 ^a^	0	19.67 ± 1.52 ^a^	212.10 ± 4.04 ^c^	0.91 ± 0.02 ^a^
	2	35.03 ± 2.85 ^ab^	351.27 ± 8.80 ^b^	0.27 ± 0.01 ^ab^	2	24.70 ± 2.19 ^a^	534.83 ± 55.57 ^a^	0.43 ± 0.06 ^b^
	4	30.66 ± 1.81 ^bc^	375.73 ± 20.21 ^b^	0.21 ± 0.05 ^ab^	4	34.70 ± 3.87 ^a^	236.37 ± 13.05 ^bc^	0.22 ± 0.02 ^bc^
	6	26.80 ± 0.66 ^c^	383.33 ± 7.64 ^b^	0.24 ± 0.02 ^ab^	6	36.10 ± 4.55 ^a^	222.70 ± 14.55 ^c^	0.19 ± 0.01 ^bc^
	8	35.80 ± 2.80 ^ab^	361.60 ± 33.13 ^b^	0.18 ± 0.02 ^ab^	8	36.90 ± 2.42 ^a^	299.03 ± 0.55 ^b^	0.19 ± 0.01 ^bc^
	10	35.86 ± 3.13 ^ab^	350.57 ± 10.28 ^b^	0.14 ± 0.02 ^b^	10	35.63 ± 3.04 ^a^	259.23 ± 1.79 ^bc^	0.18 ± 0.03 ^c^
	12	34.10 ± 2.51 ^abc^	344.87 ± 24.29 ^b^	0.24 ± 0.02 ^ab^	12	36.97 ± 3.70 ^a^	209.37 ± 21.70 ^c^	0.18 ± 0.01 ^bc^

All values are means ± SD, n = 3. Different lowercase letters (a–c) in the same column indicate statistically significant differences among system formulations (*p* < 0.05).

**Table 4:** Particle size, ζ-potential and PDI of chitosan formulations propolis-free (P − CD) at 5% (*w*/*v*).

Chitosan (*w*/*v*)	DATEM (*v*/*v*)	ζ-Potential (mV)	Particle Size (nm)	PDI	Lecithin (*v*/*v*)	ζ-Potential (mV)	Particle Size (nm)	PDI
**5**	0	27.83 ± 2.11 ^b^	395.50 ± 18.93 ^bc^	0.65 ± 0.04 ^a^	0	22.40 ± 0.98 ^c^	269.93 ± 24.93 ^cde^	0.64 ± 0.19 ^a^
	2	37.73 ± 3.20 ^a^	549.27 ± 11.15 ^a^	0.35 ± 0.05 ^c^	2	14.37 ± 1.07 ^d^	322.77 ± 5.00 ^bd^	0.23 ± 0.04 ^ab^
	4	36.63 ± 2.22 ^a^	447.03 ± 17.11 ^b^	0.24 ± 0.04 ^d^	4	25.97 ± 0.85 ^bc^	293.63 ± 5.26 ^e^	0.21 ± 0.02 ^ab^
	6	37.13 ± 3.50 ^a^	477.43 ± 2.94 ^b^	0.27 ± 0.01 ^cd^	6	37.43 ± 4.48 ^a^	385.10 ± 14.88 ^ab^	0.26 ± 0.02 ^ab^
	8	28.33 ± 0.65 ^b^	533.93 ± 10.85 ^a^	0.29 ± 0.06 ^cd^	8	27.43 ± 0.32 ^bc^	383.47 ± 11.86 ^a^	0.15 ± 0.01 ^b^
	10	36.03 ± 2.31 ^a^	377.43 ± 13.40 ^c^	0.26 ± 0.01 ^cd^	10	35.23 ± 2.25 ^a^	342.43 ± 42.39 ^abcde^	0.24 ± 0.01 ^ab^
	12	40.17 ± 1.42 ^a^	513.07 ± 38.57 ^abc^	0.46 ± 0.02 ^b^	12	31.73 ± 2.85 ^ab^	360.73 ± 7.20 ^ac^	0.23 ± 0.01 ^ab^

All values are means ± SD, n = 3. Different lowercase letters (a–e) in the same column indicate statistically significant differences among system formulations (*p* < 0.05).

**Table 5:** Identification and quantification of phenolic compounds in propolis extract by UHPLC-Orbitrap HRMS.

Phenolic Compounds	RT (min)	Confirming\Quantifier (m/z)	Concentration (µg/g)
Benzoic acid	8.9	121.02940	993.79 ± 3.79
4-Hydroxybenzoic acid	6.13	137.02442	54.06 ± 2.75
Gallic acid	2.13	169.01425	259.11 ± 7.89
Protocatechuic acid	4.26	153.01933	88.14 ± 1.96
3.4-dihydroxybenzaldehyde	5.58	137.02442	168.98 ± 3.60
Vanillic acid	7.07	167.03498	69.83 ± 3.01
Vanillin	7.57	151.04007	434.49 ± 1.26
Coumaric acid	8.26	163.04007	1139.64 ± 84.01
Caffeic acid	7.19	179.03498	2111.16 ± 158.34
Caffeic acid phenethyl ester (CAPE)	11.99	283.09758	1426.94 ± 122.39
Ferulic acid	8.54	193.05063	2868.86 ± 24.47
Chlorogenic acid	7.06	353.08781	16.87 ± 3.86
3-(4-Hydroxyphenyl) Propionic acid	7.88	165.05572	598.17 ± 6.71
Chrysin	12.18	253.05063	2641.85 ± 34.08
Apigenin	11.3	269.04555	534.56 ± 3.42
Acacetin	12.4	283.06120	882.60 ± 4.66
Apigenin 7-glucoside	9.58	431.09837	57.22 ± 1.72
Genkwanin	12.4	283.06120	406.88 ± 7.50
Luteolin	10.75	285.04046	154.82 ± 9.95
Rutin hydrate M-OH2	9.22	609.14611	1393.79 ± 55.97
Galangin	12.3	269.04555	3369.42 ± 197.01
Quercetin	10.48	301.03538	789.56 ± 3.94
Isoquercitrin	9.27	463.08820	91.50 ± 1.60
Narcissin	9.81	623.16176	286.68 ± 17.76
Isorhamnetin	11.18	315.05103	481.70 ± 1.35
Hyperoside	9.27	463.08820	91.51 ± 1.59
Kaempferide	12.35	299.05611	179.39 ± 3.32
Nicotiflorin	9.69	593.15119	81.63 ± 1.78
Astragalin	9.7	447.09328	186.05 ± 15.52
Leucoside	9.78	289.06924	128.49 ± 1.44
Naringenin	10.51	271.06120	3874.19 ± 31.78
Sakuranetin	11.85	285.07685	663.10 ± 3.25
Kuromanine	9.74	447.09328	207.00 ± 1.39

All values are means ± SD (standard deviation), n = 3. RT: retention time.

**Table 6:** Identification and quantification of phenolic compounds in propolis-loading colloidal system by UHPLC-Orbitrap HRMS.

Phenolic Compound	RT (min)	Quantifier Ion (m/z)	Concentration (µg/g)
Benzoic acid	8.84	121.02940	509.41 ± 5.19
Vanillin	7.53	151.04007	282.93 ± 1.38
Coumaric acid	8.2	163.04007	436.17 ± 5.15
Caffeic acid	7.14	179.03498	394.32 ± 12.77
Caffeic acid phenethyl ester (CAPE)	11.92	283.09758	384.80 ± 11.38
Ferulic acid	8.48	193.05063	1035.55 ± 15.41
Chlorogenic acid	7.05	353.08781	6.00 ± 0.49
Chrysin	12.13	253.05063	1862.82 ± 25.37
Apigenin	11.25	269.04555	205.63 ± 0.95
Acacetin	12.33	283.06120	241.24 ± 2.91
Apigenin 7-glucoside	9.56	431.09837	22.38 ± 1.17
Luteolin	10.71	285.04046	43.25 ± 2.48
Galangin	12.23	269.04555	1082.76 ± 32.81
Rutin hydrate M-OH2	9.13	609.14611	115.37 ± 2.27
Isoquercitrin	9.24	463.08820	39.59 ± 2.82
Narcissin	9.77	623.16176	27.12 ± 1.46
Hyperoside	9.24	463.08820	36.26 ± 3.93
Nicotiflorin	9.84	593.15119	14.15 ± 1.28
Astragalin	9.84	447.09328	30.01 ± 1.97
Naringenin	10.44	271.06120	723.11 ± 2.08
Sakuranetin	11.64	285.07685	82.73 ± 1.90

All values are means ± SD (standard deviation), n = 3. RT: retention time.

**Table 7:** Encapsulation efficiency (EE %) of individual phenolic compounds incorporated into the colloidal system.

Phenolic Compounds	Encapsulation Efficiency (%)
Benzoic acid	51.26 ± 0.80
Vanillin	65.12 ± 0.62
Coumaric acid	38.51 ± 3.93
Caffeic acid	18.83 ± 2.47
Caffeic acid phenethyl ester (CAPE)	27.24 ± 3.96
Ferulic acid	36.10 ± 0.90
Chlorogenic acid	38.69 ± 16.78
Chrysin	70.51 ± 0.25
Apigenin	38.47 ± 0.33
Acacetin	27.34 ± 0.58
Apigenin 7-glucoside	38.83 ± 3.24
Rutin hydrate M-OH2	8.29 ± 0.53
Luteolin	28.09 ± 3.56
Galangin	32.29 ± 3.39
Isoquercitrin	43.23 ± 3.03
Narcissin	9.53 ± 1.29
Hyperoside	39.71 ± 6.10
Nicotiflorin	17.35 ± 1.83
Astragalin	16.24 ± 2.02
Naringenin	18.67 ± 0.23
Sakuranetin	12.48 ± 0.42

All values are means ± SD (standard deviation), n = 3.

## Data Availability

The original contributions presented in the study are included in the article/Supplementary Materials. Further inquiries can be directed to the corresponding authors.

## Supplementary Materials

The following supporting information can be downloaded at: https://www.mdpi.com/article/10.3390/foods15183251/s1, Table S1: Analytical parameters of phenolic compounds analyzed by UHPLC Orbitrap-HRMS.

## Abbreviations

The following abbreviations are used in this manuscript:

CD	Chitosan-based colloidal delivery system
DATEM	Diacetyl tartaric acid ester of mono- and diglycerides
ASE	Accelerated solvent extraction
CLSM	Confocal laser scanning microscopy
EE	Encapsulation efficiency
WS	Walnut sausage
TBARS	Thiobarbituric Acid Reactive Substances
CUPRAC	Cupric Reducing Antioxidant Capacity
FTIR	Fourier Transform Infrared
DLS	Dynamic light scattering
TPC	Total Phenolic Content
