# Supplementary material for: Chitosan-Based Colloidal Delivery Systems as Natural Carriers for Propolis: Physicochemical Characterization and Application in Walnut Sausages
Source: Foods. 2026 Sep 15;15(18):3251. doi: 10.3390/foods15183251 (PMC13606339; doi:10.3390/foods15183251)
Supplement: Supplementary file 1 [file foods-15-03251-s001.zip › foods-4471229-supplementary.pdf]

Supplementary Material File

Table S1. Analytical parameters of phenolic compounds analyzed by UHPLC Orbitrap-HRMS

No	Phenolic Compound	RT	Parent Ion (m/z)	Confirming/Quantifier ion (m/z)	Adduct Ions	Polarity	R^2
1	Benzoic acid	8.84	121.02940	121.02940	M-H	-	0.9895
2	4-Hydroxybenzoic acid	6.83	137.02442	93.03471	M-H	-	0.9984
3	Salicylic acid	9.82	137.02442	93.03468	M-H	-	0.9907
4	3- Hydroxybenzoic acid	6.83	137.02442	93.03471	M-H	-	0.9978
5	3- hydroxyphenylacetic acid	6.71	107.05053	107.05053	M-H	-	0.9982
6	Syringic acid	7.77	197.04555	121.02975	M-H	-	0.9962
7	Gallic acid	2.12	169.01425	125.02461	M-H	-	0.9971
8	Protocatechuic acid	4.11	153.01933	109.02949	M-H	-	0.9987
9	Protocatechuic acid ethyl ester	9.08	181.05063	108.02187	M-H	-	0.9905
10	3,4- dihydroxybenzaldehyde	5.5	137.02442	136.01671	M-H	-	0.9990
11	2,4-dihydroxybenzoic acid	7.05	153.01933	67.01888	M-H	-	0.9985
12	Vanillic acid	7.03	167.03498	108.02173	M-H	-	0.9943
13	Vanillin	7.53	151.04007	108.02178	M-H	-	0.9975
14	Gentisic acid	6.38	153.01933	108.02176	M-H	-	0.9990
15	3,4- Dihydroxyphenylacetic acid	7.03	167.03498	152.01151	M-H	-	0.9943
16	Trans-Cinnamic acid	10.2	147.04515	147.04510	M-H	-	0.9910
17	Coumaric acid	8.2	163.04007	119.05027	M-H	-	0.9965
18	Caffeic acid	7.14	179.03498	135.04509	M-H	-	0.9993
19	Caffeic acid phenethyl ester	11.92	283.09758	135.04526	M-H	-	0.9904
20	Ferulic acid	8.48	193.05063	134.03751	M-H	-	0.9908
21	Sinapic acid	8.53	223.06120	193.01436	M-H	-	0.9919
22	Chlorogenic acid	7.03	353.08781	191.05624	M-H	-	0.9991
23	Quinic acid	1.01	191.05611	85.02962	M-H	-	0.9998
24	Vitamin C	0.82	142.99924	112.98865	M-H	-	0.9975
25	α-Cyano-4-hydroxycinnamic acid	9.25	188.03532	93.03470	M-H	-	0.9949
26	Catechin (cyanidanol)-p	6.35	289.07176	109.02975	M-H	-	0.9971
27	Epigallocatechin	6.28	305.06668	125.02415	M-H	-	0.9897
28	Epigallocatechin gallate	7.2	457.07763	169.01399	M-H	-	0.9970
29	Chrysin	12.14	253.05063	253.05063	M-H	-	0.9941
30	Apigenin	11.25	269.04555	117.03464	M-H	-	0.9900
31	Acacetin	12.33	283.06120	268.03717	M-H	-	0.9925
32	Rhoifolin	9.61	577.15692	431.09894	M-H	-	0.9902
33	Vicenin 2	7.82	593.15119	473.10941	M-H	-	0.9972
34	Apigenin 7-glucuronide	10.4	445.07763	269.04535	M-H	-	0.9963
35	Apigenin 7- glucoside	9.56	431.09837	269.04480	M-H	-	0.9922
36	Genkwanin	12.33	283.06120	268.03745	M-H	-	0.9961
37	Apiin	9.46	563.14160	359.07755	M-H	-	0.9903
38	Vitexin	8.68	431.09837	311.05603	M-H	-	0.9970
39	Schaftoside	8.32	563.14063	443.09949	M-H	-	0.9978
40	Rutin hydrate M-OH2	9.22	609.14611	300.02777	M-H	-	0.9910

Table S1. (continued)

41	Luteolin	10.71	285.04046	175.04039	M-H	-	0.9950
42	Luteolin-7-O- glucuronide	9.8	461.07255	285.04056	M-H	-	0.9921
43	Diosmetin	11.28	299.05611	284.03290	M-H	-	0.9909
44	Orientin	8.43	447.09328	327.05148	M-H	-	0.9970
45	Isoorientin	8.43	447.09328	327.05151	M-H	-	0.9970
46	Luteolosid	9.08	447.09290	285.03986	M-H	-	0.9910
47	Luteolin 7-rutinoside	9.0	595.16376	287.05371	M-H	-	0.9932
48	Galangin	12.25	269.04555	269.04555	M-H	-	0.9886
49	Quercetin	10.44	301.03538	151.00342	M-H	-	0.9989
50	Isoquercitrin	9.25	463.08820	300.02768	M-H	-	0.9944
51	Narcissin	9.78	623.16176	315.05139	M-H	-	0.9910
52	Quercetin 3-rutinoside 7-glucoside	7.41	771.20374	303.04846	M-H	-	0.9960
53	Isorhamnetin	11.2	315.05103	300.02780	M-H	-	0.9944
54	Hyperoside	9.25	463.08820	300.02780	M-H	-	0.9944
55	Kaempferol	10.26	285.04046	136.01686	M-H	-	0.9941
56	Afzelin	10.25	431.09837	285.04028	M-H	-	0.9968
57	Kaempferide	12.3	299.05611	284.03265	M-H	-	0.9892
58	Kaempferitrin	9.6	579.17083	431.09872	M-H	-	0.9917
59	Nicotiflorin	9.72	593.15119	285.04041	M-H	-	0.9903
60	Astragalin	9.73	447.09328	284.03281	M-H	-	0.9927
61	Tiliroside	10.67	595.14264	147.04356	M-H	-	0.9925
62	Leucoside	9.74	289.06924	153.01778	M-H	-	0.9926
63	Fisetin hydrate	9.87	285.04046	135.00900	M-H	-	0.9928
64	Naringin	9.03	579.17193	559.11502	M-H	-	0.9939
65	Naringenin	10.42	271.06120	119.05035	M-H	-	0.9908
66	Sakuranetin	11.63	285.07685	119.05032	M-H	-	0.9951
67	Narirutin	8.8	579.17193	271.06107	M-H	-	0.9929
68	Eriodyctiol	9.24	287.05501	287.05501	M-H	-	0.9937
69	Liquiritigenin	9.82	255.06628	255.06592	M-H	-	0.9808
70	Liquiritin	8.53	417.11911	255.06595	M-H	-	0.9937
71	Genistein	10.68	269.04555	269.04555	M-H	-	0.9847
72	Daidzin	8.04	415.10346	253.05191	M-H	-	0.9915
73	Formononetin	11.33	267.06628	252.04263	M-H	-	0.9904

Table S1. (continued)

74	Kuromanin	9.08	447.09328	285.03970	M-H	-	0.9896
75	Ellagic acid	9.44	300.99899	300.99872	M-H	-	0.9984
76	Esculin hydrate	6.19	339.07216	177.01930	M-H	-	0.9983
77	Phloridzin	9.3	435.12967	273.07648	M-H	-	0.9911
78	Rosmarinic acid	9.49	359.07724	161.02451	M-H	-	0.9979
79	Glabridin	12.63	323.12888	135.04533	M-H	-	0.9972
80	Arbutin	0.8	271.08233	108.02180	M-H	-	0.9948
81	Emodin	13.35	269.04555	225.05573	M-H	-	0.9922
82	Etoposide	9.86	383.11053	229.04857	M-H	-	0.9868
83	3-(4-Hydroxyphenyl) propionic acid	9.26	165.05572	108.02195	M-H	-	0.9927

RT: Retention time; Quan Peak (Parent Ion (m/z)); Confirming Ions (Fragment Ions (m/z)); R^2: Coefficient of determination
